# Editorial: Immunogenicity of Proteins Used as Therapeutics

**DOI:** 10.3389/fimmu.2020.614856

**Published:** 2020-10-29

**Authors:** Zuben E. Sauna, Susan M. Richards, Bernard Maillere, Elizabeth C. Jury, Amy S. Rosenberg

**Affiliations:** ^1^ Hemostasis Branch, Division of Plasma Protein Therapeutics, Office of Tissues and Advanced Therapies, Center for Biologics Evaluation and Research, Food and Drug Administration, Silver Spring, MD, United States; ^2^ Translational Medicine and Early Development, Sanofi Research and Development, Sanofi, Framingham, MA, United States; ^3^ Université de Paris-Saclay, CEA, INRAE, Département Médicaments et Technologies pour la Santé, SIMoS, Gif-sur-Yvette, France; ^4^ Centre for Rheumatology, Division of Medicine, University College London, London, United Kingdom; ^5^ Laboratory of Immunology, Division of Biotechnology Product Review and Research 3, Office of Biotechnology Products, Center for Drugs Evaluation and Research, U.S. Food and Drug Administration, Silver Spring, MD, United States

**Keywords:** therapeutic proteins, immunogenicity, drug development, drug safety, immune tolerance

“*And a little child shall lead them*”

Nowhere is immunogenicity of therapeutic proteins of greater concern or impact than in children who potentially face a lifetime of treatment for chronic disease. This is brought into focus by three publications [Gress et al.; Desai et al.; Scott and Pratt].

Gress et al. make clear that the outcome for children with Inflammatory Bowel Disease (IBD) who lose efficacy due to Anti-Drug Antibodies (ADA) requires recurrent surgeries and eventually bowel resection and colostomy creation. While this outcome is hard for adults with IBD, it is devastating to children. To allow better Tumor Necrosis Factor (TNF)-inhibitor mediated control of IBD, this article suggests: 1) Keeping levels of TNF inhibitors over a critical level (which appears to preclude ADA in most patients) using therapeutic drug monitoring (TDM). 2) Accessibility to tests detecting ADA at the earliest time point allowing consideration of therapeutics that will prevent further escalation of ADA. 3) Development of immune tolerance induction protocols. The addition of co-administered immune suppressant agents (e.g. methotrexate, azathioprine) at the introduction of the biological therapy has been shown in numerous studies to reduce ADA development and potentially induce tolerance to the biological therapy. Whether patients co-administered these agents are then truly tolerant to the TNF antagonist has not been formally evaluated. Moreover, the duration of the dual immune suppressive therapy necessary for insuring tolerance to the biological component should be studied. Key to these approaches is the ability to readily test, at no or low cost, patient samples for levels of the TNF antagonist as well as ADA.

Desai et al. present an elegant approach to addressing ADA that neutralize a life-saving enzyme replacement therapy (ERT), recombinant human alpha glucosidase (rhGAA). In a subpopulation of Infantile Pompe Disease (IPD) patients that lack endogenous GAA, immune tolerance induction (ITI) is essential to a favorable patient outcome. This protocol, in which a short course of methotrexate, rituximab, and IVIG are given concurrently with initial doses of ERT proved highly tolerogenic. In the absence of this protocol these IPD patients would lose motor milestones and die of cardiopulmonary failure. Patients treated with ITI protocol recovered their B cell populations over several months and mounted vaccine responses, demonstrating the transient nature of the safety concerns. A case study by Gupta et al. demonstrated the effectiveness of early ITI in a prenatally diagnosed patient with IPD who started treatment immediately after birth. This is the first report of successful ITI at such an early age. Critically, if patients were not treated concurrently with initial dosing of the ERT and did develop life threatening ADA, the prophylactic regimen failed to eliminate antibody producing plasma cells. Thus, adding a proteasome inhibitor (bortezomib) to the regimen, resulted in dramatic diminution and elimination of the ADA, facilitating immune tolerance to ERT and allowing patients to recover their motor milestones (1). Finally, Scott and Pratt provide a description of the onerous ITI protocol following immune response to coagulation factor replacement therapy which hits children the hardest.

Anticipating an ADA response to therapeutic proteins remains the holy grail. In the ideal world, reliable non-clinical methods would predict immune responses early in clinical development. There is an unmet need for two classes of tools that assess the likelihood that a drug will generate a significant immune response. The first is a suite of in silico and *in vitro* methods that can assess immunogenicity during drug development prior to the clinical use of a protein drug. However, as most approved therapeutic proteins have some degree of ADA formation, there is also a need for assays that can be used in the clinic so that physicians can make informed decisions for individual patients with respect to either tempering the immune response or seeking out alternative treatment options. The report by Baker et. al. on Alemtuzumab illustrates these key points. Alemtuzumab is a humanized monoclonal antibody that is paradoxically among the most immunogenic of monoclonal antibody products; humanization does not render a protein fully human and even fully human proteins may elicit ADA. The experience with Alemtuzumab is not surprising because although immune responses are broadly predicated on the concept of “self” and “non-self”, immuno-biology is nuanced and complex (as evidenced by the challenge posed by autoimmune diseases). In this issue, two reviews from Vaisman-Mentesh et. al. and Nabhan et al. provide an excellent introduction to the molecular and cellular mechanisms involved in the generation of ADAs. Additionally, Fitzpatrick et al. provide a fascinating overview of the role of recombinant Fc multimers in immune tolerance induction and suggest that monoclonal antibodies used for treatment of cancer and autoimmunity, may mediate tolerogenic effects. This could be mediated by immune complex formation or by antibodies coating target cells, triggering multiple mechanisms including antibody dependent cell-mediated cytotoxicity, complement activation and regulatory T cell expansion, that reset immune homeostasis in an antigen independent manner. Thus, an understanding of the underlying biology is essential for designing tools to assess and circumvent immunogenicity.

Following up on the concept that immunogenicity goes beyond strict self/non self-discrimination, two comprehensive surveys of the current state of the art with respect to non-clinical immunogenicity assessments are provided by Jawa et. al. and Meunier et. al.. Jawa et al. list technical approaches to assess immunogenic potential and provide a very useful discussion of integrating immunogenicity data in regulatory submissions. These two articles also provide mechanistic context for immunogenicity and of the assays being discussed. The identification of T cell epitopes in biopharmaceuticals reveals multiple mechanisms leading to T cell activation, ADA response or regulation (Meunier et al.). Variability, diversity, and joining (VDJ) recombination of antibodies and somatic mutations resulting from affinity maturation processes contribute to making non germline sequences, which could be recognized as new T cell epitopes (neoepitopes). However, though not mutated, therapeutic protein counterparts of endogenous proteins such as recombinant hormones, growth factors and cytokines can elicit CD4 T cell responses and elicit ADAs. The basis for their immunogenicity may pertain to the insufficient expression of their endogenous counterpart in the body and thus failure to induce central tolerance. Functional T cells therefore escape from negative thymic selection by insufficient levels or affinity for self-antigens leading to failure of deletion or induction of anergy, although they are specific for self-sequences and not neoepitopes. These escaped T cells may be activated in the periphery by infusion of the therapeutic proteins (Meunier et al.). Both articles (Jawa et. al.; Meunier et al.) also provide an important caveat to the use of T-cell proliferation assays to assess immunogenic potential. Although T cell activation is essential for high titer, class switched, affinity matured antibody response, they do not always promote ADA responses and might differentiate instead into regulatory IL-10 secreting T cells (Tr1), with IL-10 being able to dampen activation of effector T cells (Jawa et. al.; Meunier et. al.).

In addition to these broad surveys of the literature, Karle provides an in-depth critical discussion of an important emerging technology. The MHC-associated peptide proteomics (MAPPs) assay is technically challenging but allows the direct identification of the naturally processed peptides derived from biopharmaceuticals displayed by HLA class II molecules on DCs. MHC class II molecules are immunopurified and the bound peptides are eluted by acidic treatment and sequenced by liquid chromatography-mass spectrometry. Karle describes how MAPPs is used to investigate immunogenicity risk of biopharmaceuticals but also to evaluate their capture by antigen presenting cells and the impact of post-translational modifications, folding, and aggregation on peptide presentation. The article provides a useful summary of all studies that have applied the MAPPs technology to therapeutic proteins, and by comparing multiple studies using the same therapeutic, shows that the technique is reliable and renders consistent results. Unfortunately, as with all non-clinical estimates of therapeutic protein immunogenicity, the clinical significance of the results remains the critical unknown.

Data sets of peptides eluted from MHC molecules in MAPPs assays are rapidly growing and are being leveraged to improve in silico algorithms used in immunogenicity assessments. Peptide-MHC engagement is a necessary step in the initiation of an immune response to therapeutic proteins. Thus, algorithms that predict peptide-MHC binding affinities have become an initial rapid and inexpensive screen for potential immunogenicity. One reason why such algorithms overestimate immunogenicity risk is that not all potential high-affinity peptides are actually generated by the protease machinery of antigen presenting cells. By directly identifying therapeutic protein-derived peptides on MHC molecules, the results of MAPPs assays report on both peptide processing and presentation. Thus, in this Research Topic, Barra et al. report an artificial neural network (ANN) model, trained to predict T cell epitopes. The algorithm presented by Barra et al. joins multiple in silico methods that are freely available to estimate immunogenicity risk based either on HLA binding affinity or on MAPPs data.

The profusion of freely available tools is a double-edged sword for those not familiar with the computational and statistical methods used. For end users of such algorithms, Paul et. al. introduce the need of rigorous benchmarking to compare the different methods of identification of T cell epitopes and provide quantitative data to point out the benefits and insufficiencies of each method (Paul et. al.). While some algorithms have become increasingly accurate at specific tasks, e.g. estimating peptide-MHC affinity or which peptides will be presented by MHC molecules, the ability to predict clinical immunogenicity remains a key challenge. Predicting immunogenicity for biotherapies using patient and drug-related factors is attractive but no robust method has as yet been developed. With the growing ability to collect massive amounts of data, machine learning algorithms could identify predictive variables. Two studies applied machine learning models to predict ADA status (Duhazé et al. and Waddington et al.) utilized clinical data collected in the multi-cohort of autoimmune diseases treated with biotherapies from the ABIRISK consortium. Duhazé et al. evaluated the predictive power of a custom-built machine learning model, the random survival forest model (2), for predicting the occurrence of anti-drug antibodies. The approach provided a good predictive accuracy and outperformed current methods, although validation in larger cohorts is needed.

In the discussions thus far, the MHC repertoire is as an important parameter in assessing immunogenicity because foreign-peptide-MHC binding is a necessary (albeit not sufficient) step in the immune response leading to ADAs. However, the MHC genes are the most polymorphic in the human genome and occur at different frequencies in various human subpopulations. This creates a problem in putting together a suitable real or virtual cohort for immunogenicity assessments that is representative of the population of interest with respect to distribution of MHC variants. McGill et al. have addressed this challenge by developing an algorithm, SampPick, that permits the selection of a cohort of subjects that matches a population MHC distribution.

As discussed earlier, assessment of the potential for immunogenicity represents an unmet need not only for biomolecules in the early stages of development but also in the clinic. Most approved therapeutic proteins do exhibit various levels of immunogenicity and their clinical use would benefit from accurate, reproducible and clinically meaningful measurements of ADAs and TDM. Atiqi et. al., Mehta and Manson, and Gress et al. describe the scope of the problem using the example of TNF-Inhibitors. These medications have revolutionized the management of rheumatoid arthritis and other diseases but only a small proportion of patients maintain long-term clinical response (3, 4). Boyer-Suavet et al. describe how the presence of neutralizing anti-rituximab antibodies is similarly associated with disease relapse. Selection of, and switching between, biologics is mainly empirical, experiential, and not evidence-based. While it is broadly acknowledged that immunogenicity is one of the main reasons for loss of therapeutic efficacy (secondary failure) the field is beset by challenges. ADA identification is technically difficult and not standardized, making interpretation of immunogenicity data and application in the clinic almost impossible (Mehta and Manson). However, Lallemand et al. reported on the potential of using a highly sensitive reporter gene assay to quantify both an anti-VEGF ADA and the therapeutic drug activity to monitor responder vs. non-responder patients. Overall, longer term information is needed to determine the utility of these approaches. This Research Topic, however, does also offer potential strategies to overcome some of these problems (Kharlamova et al.) and novel assays that may be more reliable (Kharlamova et al.).

In keeping with our increased understanding of the complexity and diversity of immune responses four papers provide a glimpse into the application of new but rapidly emerging fields to therapeutic protein immunogenicity. Fu et al. address biotherapeutic immunogenicity in the context of the orchestrated function of highly differentiated T and B cells, including follicular helper CD4 T cells and germinal center B cells, for the optimal generation of antibody responses. They suggest that understanding the cellular and molecular mechanisms mediating the antibody responses against therapeutics could lead to novel strategies to reduce their immunogenicity. Kishimoto describes a promising approach to antigen targeted immune tolerance induction to prevent the formation of ADAs across a wide variety of biologics. Antigen-targeted tolerance is induced in several experimental animal models (haemophilia A, inflammatory arthritis, and Pompe disease) by the incorporation of rapamycin in nanoparticles in the presence of therapeutic antigen at the time of administration (ImmTOR). The problematic immunogenicity issues pertaining to recombinant immunotoxins (chimeric proteins consisting of a targeting element such as a Fv antibody region bound to a toxin) are reviewed by Mazor and Pastan. These therapies have potential for use in a wide variety of diseases and have been tested and approved in cancer. This review outlines the strategies used to mitigate the immunogenicity of immunotoxins which has a major impact on the efficacy of these promising drugs. Finally, Waddington et. al. have presented original research that applies the rapidly expanding field of serum metabolomics to therapeutic protein immunogenicity. Their study shows that serum metabolites are a promising biomarker for early identification of ADA development in MS patients treated with IFNβ and could provide novel insights into mechanisms of immunogenicity.

Significant effort is spent during clinical development in commissioning robust, specific and sensitive ADA assays that are validated to support clinical studies for product approval. However, longer term evaluation is needed to facilitate translatability to routine clinical practice that can impact patient care. ADA status (ADA-positive vs. ADA-negative) is just the first tier for assessment. Clinical relevance of ADA on PK, PD biomarkers and safety (e.g. infusion associated reactions, hypersensitivity) is more evident when assessed in the context of either quartile or tertile ADA titer groups as well as evaluation of neutralizing activity. Determining relevance of ADA on efficacy parameters is complex as the measured clinical outcomes are often distal from the site of drug action and more time is needed to observe the consequences of diminished drug efficacy due to ADA. This outcome is now seen with the class of TNF-alfa inhibitor drugs and enzyme replacement therapies for some lysosomal storage diseases. Determining a clinically relevant ADA titer can be challenging and therefore, TDM is an alternative approach as data indicate that keeping levels of some biological therapeutics above a threshold value diminishes the probability of ADA generation and improves patient outcome. Post marketing commitments and requirements are mechanisms for obtaining long term data to address practical questions. However, there are challenges with this approach. After approval or licensure, such testing is usually done in a CLIA or other regulated clinical diagnostic lab. The lack of standardized assays to assess drug concentrations and ADA further complicates interpretation of results and identification of the reason for the loss of response. Companies, in collaboration with health care providers and insurers, have a responsibility to address in clinical practice the key questions regarding ADA and therapeutic drug monitoring, provide actionable answers, and establish means to make such testing readily available to ensure patients receive efficacious drug dosing. Robust analyses of the economic impact of ADA may motivate payers to support these efforts.

## Author Contributions

All authors contributed to the article and approved the submitted version.

## Funding

Research conducted in the laboratory of ZS is funded by FDA intramural grants.

## Disclaimer

This article reflects the views of the author (AR) and should not be construed to represent FDA’s views or policies.

## Conflict of Interest

SR is an employee of Sanofi and owns stock in the company.

The remaining authors declare that the research was conducted in the absence of any commercial or financial relationships that could be construed as a potential conflict of interest.
